# Lateral migration of large sedimentary bodies in a deep-marine system offshore of Argentina

**DOI:** 10.1038/s41598-021-99730-x

**Published:** 2021-10-13

**Authors:** Adam Kirby, Francisco Javier Hernández-Molina, Sara Rodrigues

**Affiliations:** Department of Earth Sciences, Royal Holloway University of London, Egham, Surrey, TW20 0EX UK

**Keywords:** Geology, Geomorphology, Geophysics, Sedimentology

## Abstract

Contourite features are increasingly identified in seismic data, but the mechanisms controlling their evolution remain poorly understood. Using 2D multichannel reflection seismic and well data, this study describes large Oligocene- to middle Miocene-aged sedimentary bodies that show prominent lateral migration along the base of the Argentine slope. These form part of a contourite depositional system with four morphological elements: a plastered drift, a contourite channel, an asymmetric mounded drift, and an erosive surface. The features appear within four seismic units (SU1–SU4) bounded by discontinuities. Their sedimentary stacking patterns indicate three evolutionary stages: an onset stage (I) (~ 34–25 Ma), a growth stage (II) (~ 25–14 Ma), and (III) a burial stage (< 14 Ma). The system reveals that lateral migration of large sedimentary bodies is not only confined to shallow or littoral marine environments and demonstrates how bottom currents and secondary oceanographic processes influence contourite morphologies. Two cores of a single water mass, in this case, the Antarctic Bottom Water and its upper interface, may drive upslope migration of asymmetric mounded drifts. Seismic images also show evidence of recirculating bottom currents which have modulated the system’s evolution. Elucidation of these novel processes will enhance basin analysis and palaeoceanographic reconstructions.

## Introduction

Clinoforms are common in marine environments. Their relief ranges from tens of meters in deltaic and shallow marine environments up to 500 m thickness along shelf edges, whilst clinoforms on continental slopes (continental margin clinoforms) can range from 500 to 1000 m in thickness^1^. Clinoforms are defined as sloping surfaces that form free from disturbance beneath the wave base and typically consist of fine grained, thinly and evenly bedded sediment^1,2^. They are sensitive to sea level changes, particularly in shallower environments, and typically accrete in a basinward direction^1^. The angle of inclination, sediment supply, composition and vertical accommodation space between the wave base and the flat seafloor determine clinoform length^1,3^. In deep marine settings, some recent studies have shown that deposits share similar stacking patterns to clinoforms, though they result from different processes such as lateral migration, these include downslope channel-levee systems, mixed turbidite-contourite systems controlled by downslope and alongslope processes^4,5^, giant mounded contourite drifts controlled by bottom currents^1,6^, subaqueous sand dunes controlled by internal solitary waves^7^, and deep marine channels where bottom currents, turbidity currents, and internal waves/tides interact^8^.

Bottom currents play a significant role in shaping continental margins and abyssal plains^9^. They generate a range of depositional (drifts), erosional (channels), or mixed features referred to as ‘contourites’, which typically appear in margin-parallel orientations, and together form a ‘Contourite Depositional System’ (CDS)^10^. Asymmetric mounded drifts, classified as giant, elongated drifts^6,9^ commonly appear in slope-adjacent and open marine environments but these features and the mechanisms that form them remain poorly understood^11^. The Argentine continental rise hosts an extensive, buried CDS with a large asymmetric mounded drift that shows remarkable upslope migration. The present study interpreted the main morphological features and sedimentary stacking pattern of this CDS. The research also sought to interpret major upslope sedimentary migration patterns (> 500 m relief) occurring in this deep marine setting. The migration, though directed upslope, has a similar pattern to that of clinoform progradation but appears to be driven by bottom currents and secondary oceanographic processes associated with the pycnocline. This paper presents a model for the evolution of the system that generally explains how bottom currents can induce lateral upslope migration of sedimentary bodies.

### Regional geologic and oceanographic framework

**Figure 1:**
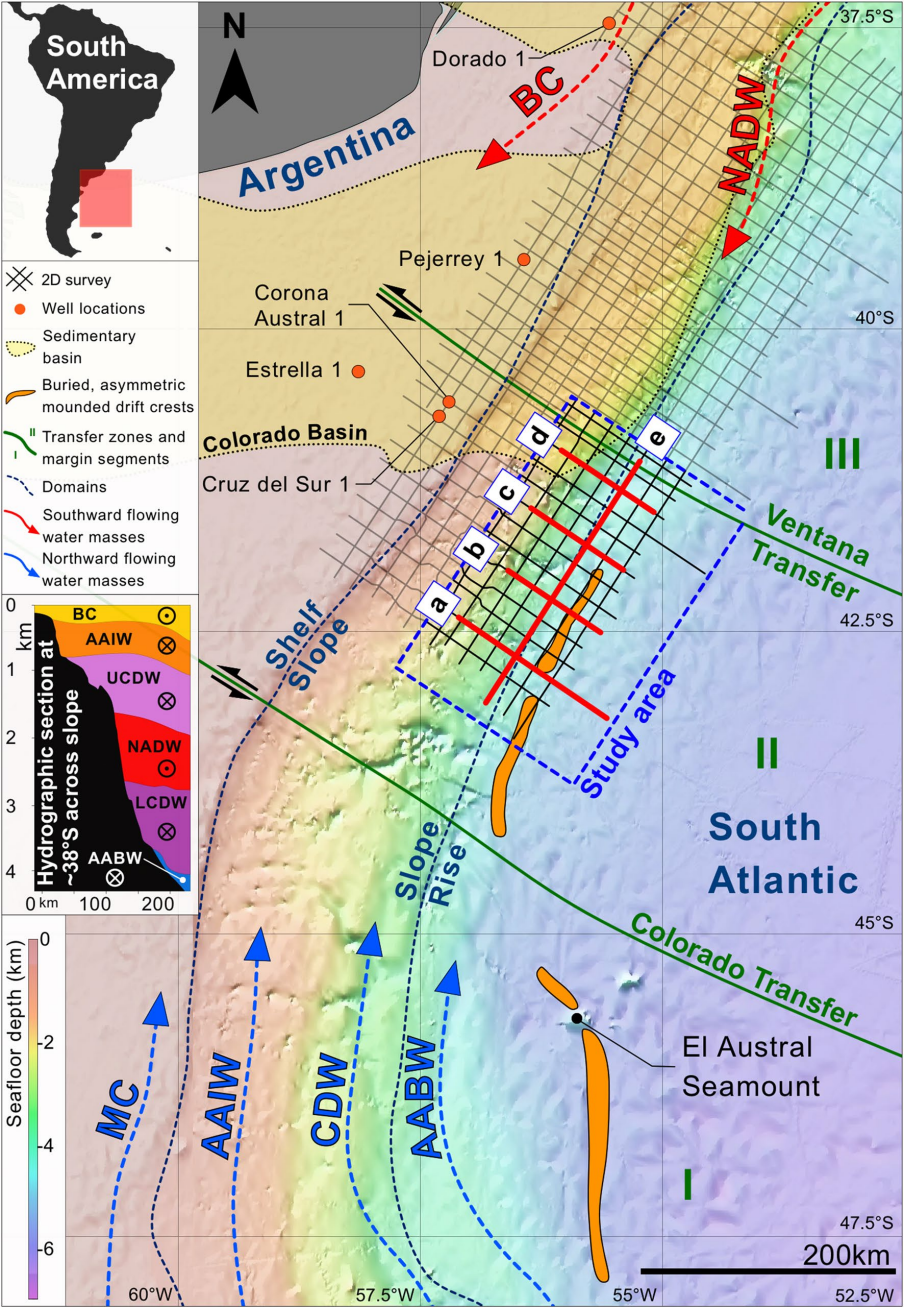
Regional bathymetric map from Tozer et al. (2019)^16^ showing the study area, domains, dataset, seismic profile figures (**a**–**e**), and buried drift crests. Arrows indicate flow directions of surface (BC = Brazil Current, MC = Malvinas Current), intermediate (AAIW = Antarctic Intermediate Water), deep (NADW = North Atlantic Deep Water, CDW = Circumpolar Deep Water), and bottom (AABW = Antarctic Bottom Water) waters^17^. Hydrographic section adapted from Hernández-Molina et al. (2010)^18^ after Piola and Matano et al. (2001)^19^; sedimentary basins, rift segments and fracture zones adapted from Franke et al. (2007)^12^. The figure was generated using Pixelmator Pro 2.1.3 Coral (https://www.pixelmator.com/pro/).

Situated between 35° and 48° S, the Argentine margin is a classic passive, segmented rift margin underlain by a volcanic basement (Fig. 1)^12^. It extends over 1500 km in a generally NE-SW direction, spans a width of 50–300 km, and covers a ~ 700,000 km^2^ area with an average slope gradient of 2°^13^. Previous research has defined the margin as consisting of four, ~ 400 km long segments (I–IV) separated by major transfer zones^12^. The margin hosts a total of six sedimentary basins^14^. A buried CDS consisting of two asymmetric mounded drifts occurs in segments I and II^15^. This research addresses a previously uninterpreted portion of the northernmost drift at a locality to the south of the Colorado Basin and situated below the present-day lower slope and continental rise at > 3.5 km water depth (Fig. 1).

Numerous water masses flow along the Argentine margin making it one of the most dynamic oceanographic regions in the world (Fig. 1)^19,20^. Water masses include the surficial Brazil and Malvinas Currents and the intermediate (< 1 km water depth) Antarctic Intermediate Water. Deep waters (1–3.5 km water depth) include the Circumpolar Deep Water (CDW) consisting of the Upper and Lower Circumpolar Deep Water (UCDW and LCDW), and the North Atlantic Deep Water (NADW) that flows between them (Fig. 1). Bottom waters (> 3.5 km) are dominated by the Antarctic Bottom Water (AABW), which becomes partially trapped in the Argentine Basin and forms a cyclonic gyre between 3.5 and 4 km water depth^19^. Pycnoclines separating successively deeper water masses are formed with increasing density due to lower temperatures and/or higher salinity^10^. These generally deepen to the north in the Argentine Basin but form at their shallowest depths across the CDS^20^.

## Dataset and methods

This study interpreted ~ 40,000 km of 2D depth-migrated multichannel seismic reflection profiles acquired in 2017 and 2018 by Spectrum (now TGS). Separated by 10–20 km, the profiles have a maximum vertical resolution of ~ 9 m and maximum frequency of 75 Hz (Fig. 1). Streamers ran 15 m deep and 12,000 m in length while the source was 8 m deep with a volume of 4230 in^3^. Shot intervals were 25 m and the sample rate was 1 ms.

Data were subjected to a pre-stack time migration and a pre-stack depth migration with full waveform inversion and broadband processing. Anisotropic ray-based Kirchhoff migration was used to migrate the seismic data from the time (ms) to the depth (m) domain. After derivation of the water column velocity profile, the original velocity model used the available root mean squared velocity information extracted from the time data. The model was then subjected to three iterations of ray-based inversion to minimize the velocity error. The method began by stripping shallow layers with iterative updates of long wavelength velocity and then progressively incorporating shorter wavelengths for deeper layers.

Several wells (Fig. 1) provided additional information including key horizons for the Upper Cretaceous and upper Eocene, which are based on a revised chronostratigraphic framework^21,22^ originally developed by Petrobras Argentina.

The analysed drift occurs in the southwestern sector of the dataset and was interpreted at three spatial scales spanning from seismic units to seismic facies^23^ and following conventional methods for seismic interpretation^2,24^.

### Seismic analysis

**Figure 2:**
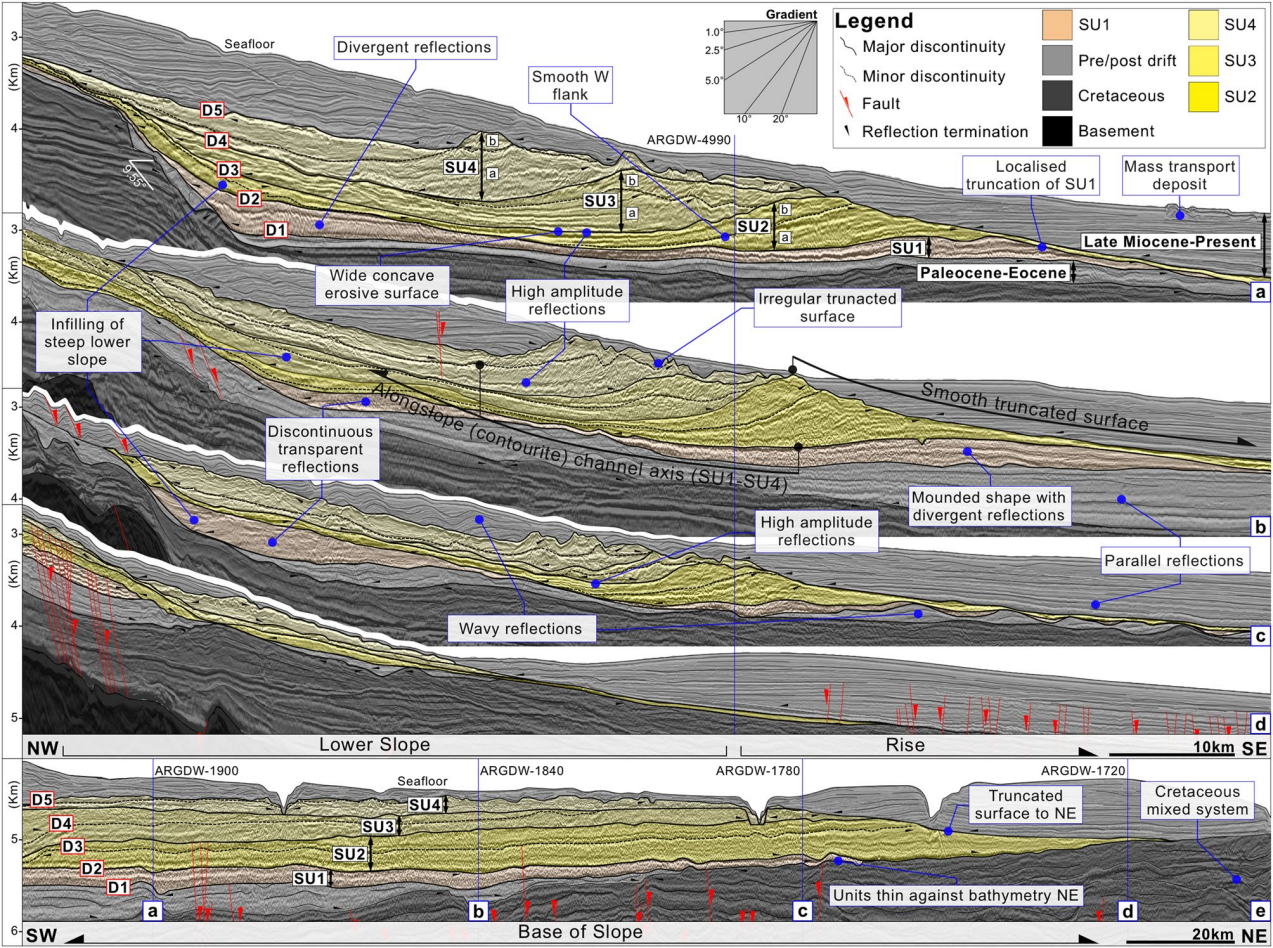
Interpreted seismic crosslines (**a**–**d**) and inline (**e**) profiles detailing discontinuities (D1–D5), seismic units (SU1–SU4), and sub-units (**a**/**b**). The figure was generated using Pixelmator Pro 2.1.3 Coral (https://www.pixelmator.com/pro/).

**Figure 3:**
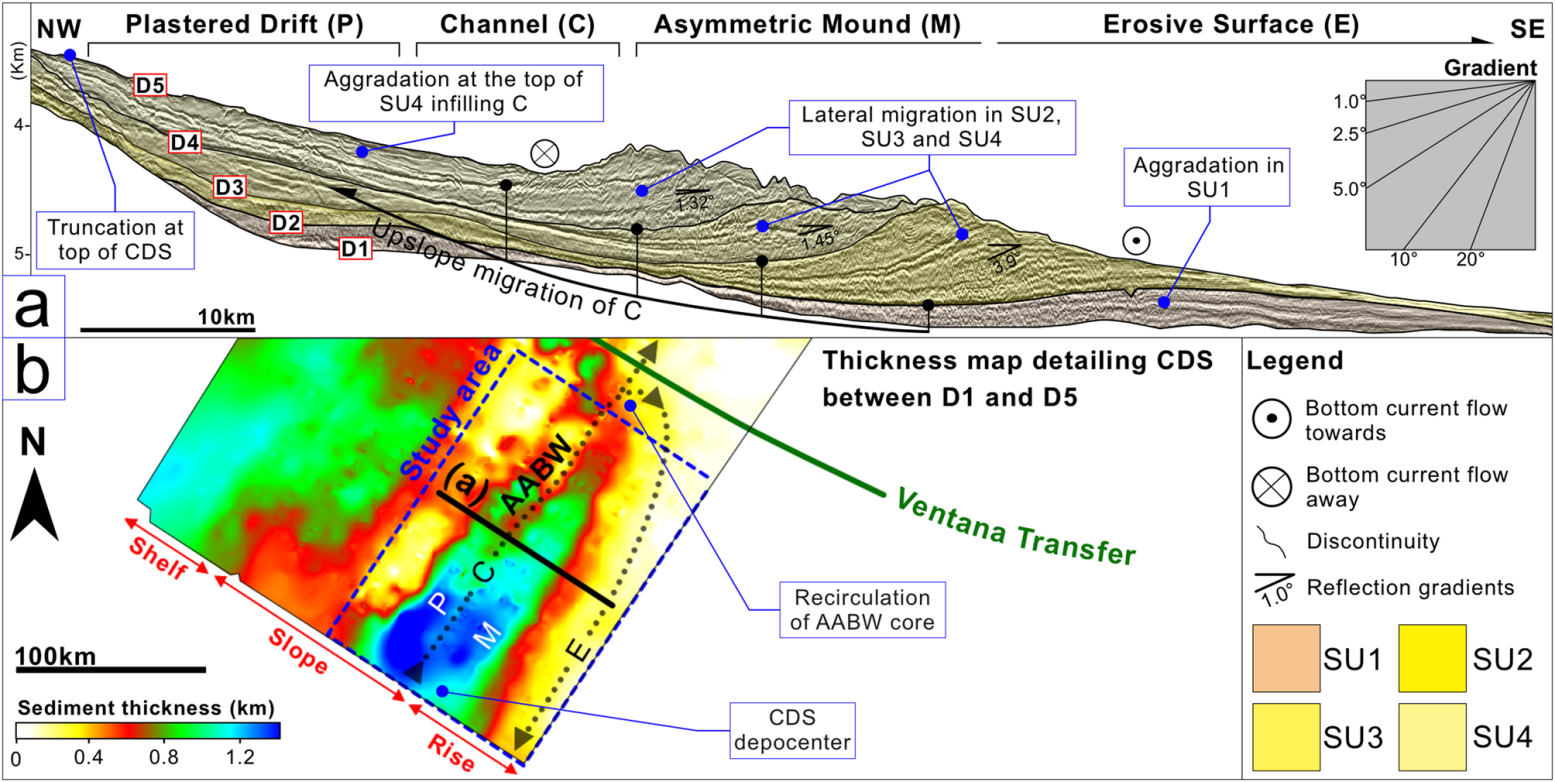
(**a**) Seismic profile detailing morphological features (P, C, M, and E), sedimentary stacking pattern, reflection gradients, and AABW cores; (**b**) map detailing the depocenter distribution and sedimentary thickness of the CDS. The figure was generated using Pixelmator Pro 2.1.3 Coral (https://www.pixelmator.com/pro/).

Five major discontinuities (D1–D5) bound four seismic units (SU1–SU4). With the exception of SU1, each of these consisted of two sub-units (a/b) (Fig. 2A). D1 represents the base of the analysed succession and corresponds to the upper Eocene (~ 34 Ma) well top (Table 1). The entire deposit is < 1250 m thick (Fig. 3B) and spans > 100 km width in the southernmost part of the study area. Across the rise, it extends beyond the survey area to the southwest and gradually thins against the underlying bathymetry to the northeast over the course of a ~ 250 km distance. The northernmost ~ 60 km of the deposit is truncated by a smooth surface against which the deposit terminates (Fig. 2D,E). All units exhibit sub-parallel and divergent reflections, while SU1 also shows discontinuous transparent reflections (Fig. 2B,C).

**Table 1:**
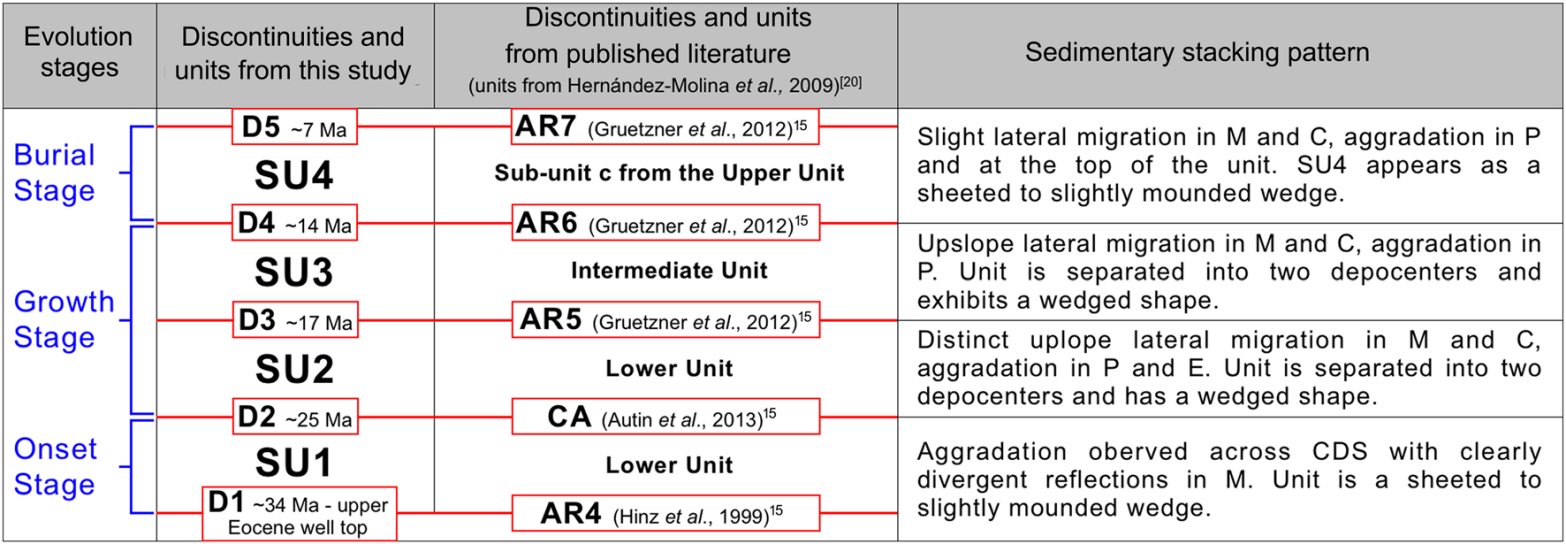
Table showing evolutionary stages, the main seismic unit and discontinuities correlated with well formation tops and published records, and a summary of the sedimentary stacking pattern. The table was generated using Pixelmator Pro 2.1.3 Coral (https://www.pixelmator.com/pro/).

SU1 is bounded at its base by D1 and capped by D2. D1 shows faint evidence of erosion and locally terminates against the steep (< 9.6°) lower slope and pre-existing relief to the northeast (Fig. 2A). Internal reflections show a range of low- to high-amplitudes. These onlap D1 and toplap D2 to the northeast and in both landward and basinward directions. The unit shows local truncation in basinward areas (Fig. 2A). Its wedge shape in profile pinches out to the northeast and exhibits a sheeted to slightly mounded configuration with a maximum thickness of < 500 m. Sediment is localised at the base of the lower slope and basinward on the rise. Reflections for this unit subtly diverge towards the depocenters (Fig. 2A,B).

SU2 is bounded basally by D2 and is capped by D3. D2 is a regional discontinuity that frequently truncates SU1. Internal reflections range from low to high amplitude. These onlap the basal surface extending beyond SU1 landwards and in a northeasterly direction (Fig. 2A,E). They show local toplap and are frequently truncated by D3. This unit includes a < 510 m thick landward depocenter with a sheeted to slightly mounded configuration plastered against the lower slope, and a more distal, < 800 m thick depocenter that displays a large asymmetric mounded configuration (Fig. 2A,B). Both of these form above the depocenter localities described within SU1. The mounded sedimentary body features a steep, truncated eastern flank related to a smooth alongslope surface. The truncation resembles that exhibited by SU1 but appears more extensive (Fig. 2A,B). The mound body also includes a smoother, more depositional western flank with a gradient of up to < 3.9° and having a mean value of 3.2° (Fig. 3A). A wide and concave surface divides these two deposits within the lower slope and rise. The surface marks out the presence of a wide and shallow alongslope channel which is oriented northeast-southwest, shows lateral continuity (see Fig. 2A–C), and is associated with high amplitude reflections (HAR’s) (Fig. 2A,B). The channel exhibits a mean width of 23.7 km, a depth of 198 m, and < 340 m of aggradation. SU2 occurs ~ 19.6 km landward of SU1 and forms a wedge shape where it thins against the bathymetry to the northeast (Fig. 2E). The unit shows an aggradational sedimentary stacking pattern except within the distal depocenter (Fig. 2B), where internal reflections show a distinct ~ northwesterly lateral migration, moving upslope a mean distance of 11.7 km, and with reflection gradients reaching 1.78° (Fig. 3A).

SU3 is bounded basally by D3 and capped by D4. D3 is a prominent regional discontinuity appearing within the lower slope and rise. Intermediate- to high-amplitude internal reflections onlap D3 landward, basinward, and to the northeast (Fig. 2E). This unit is truncated at its top in basinward and northeasterly areas. The same two depocenters described in SU2 continue to develop in SU3. The landward depocenter exhibits a sheeted structure and reaches a thickness of < 500 m, and the more distal depocenter reaches thicknesses of < 700 m and exhibits a mounded shape, it shows a landward shift of ~ 10.9 km and buries the mound from SU2 (Fig. 2A–C). The mound has a westerly flank with a gradient ranging from 2.5° to < 3.2°. The alongslope channel between these two depocenters appears as a series of high amplitude reflections, this feature spans a width of 18 km, incises to depths of 193 m and also exhibits a landward shift of ~ 10.9 km (Fig. 2A–C). Internally the mounds sedimentary stacking pattern reveals a northwesterly (upslope) lateral migration of 7.1 km where reflection gradients reach 1.3° (Fig. 3A). Overall, SU3 assumes a wedged shape that thins to the northeast.

SU4 is bounded at its base by D4 and capped by D5. D4 is a prominent discontinuity showing evidence of erosion. Low- to high-amplitude internal reflections onlap D3 and D4 landward, basinward, and alongslope. D5 represents an irregular surface which mainly truncates SU4 and locally truncates SU3 (Fig. 2B). This surface connects to the aforementioned erosive surface described above, whose smoother surface predominantly truncates SU1 and SU2 (Fig. 2A–D). During the deposition of SU4, the alongslope channel is infilled by a single, < 810 m thick sheeted deposit that appears most evidently in sub-unit b (Fig. 2A). This unit exhibits a wedged shape with a single depocenter that thins against the bathymetry to the northeast (Fig. 2D,E). SU4 lies ~ 9.1 km landward of SU3 and the former buries the latter. SU4 also exhibits minor lateral migration in sub-unit a but aggradation in sub-unit b (Fig. 3A).

### Morphosedimentary features

The seismic units described here include four prominent and related alongslope features, two of which are depositional and two of which are predominantly non-depositional and/or erosive (Fig. 3A,B). From the lower slope to the rise, these features include a plastered drift (P) based on the criteria given in Faugères et al. 1999^23^, a contourite channel (C)^9,10^, a large asymmetric mound (M), and a smooth alongslope erosive surface (E). The plastered drift (P) dips seaward and abutts the lower slope between ~ 3400 and 5000 m depth. It also shows divergent reflections towards its centre. The contourite channel (C) appears as a large feature spanning a 37.8 km width, it is situated at ~ 4491–5440 m depth. From SU1 to SU4, the channel migrates upslope by a mean distance of 13.1 km (Fig. 2B). The asymmetric mound (M) rests at ~ 4379–5650 m depth and spans a width of ~ 67.3 km. The smooth alongslope erosive surface (E) occurs at ~ 4506 to < 5800 m depth and is associated with the basinward flank of M (Figs. 2B and 3A).

### Chronology and evolutionary stages

In addition to the aforementioned well horizons which correspond to the Upper Cretaceous and upper Eocene (D1), all discontinuities were cross-referenced with predefined boundaries from published sources describing the Argentine Margin (Table 1). D1 corresponds to AR4 (~ 34 Ma) from Hinz et al. (1999)^25^, while D2 is correlated with the boundary CA (~ 25 Ma) from Autin et al. (2013)^26^. D3, D4 and D5 match AR5 (~ 17 Ma), AR6 (~ 14 Ma) and AR7 (~ 7 Ma) respectively from Gruetzner et al. (2012)^15^. The seismic units coincide with a regional stratigraphic framework proposed by Hernández-Molina et al. (2009)^20^ that consists of a lower, intermediate, and upper unit. SU1 and SU2 are coeval with the lower unit, SU3 with the intermediate unit, and SU4 with sub-unit c from the upper unit (Table 1).

**Figure 4:**
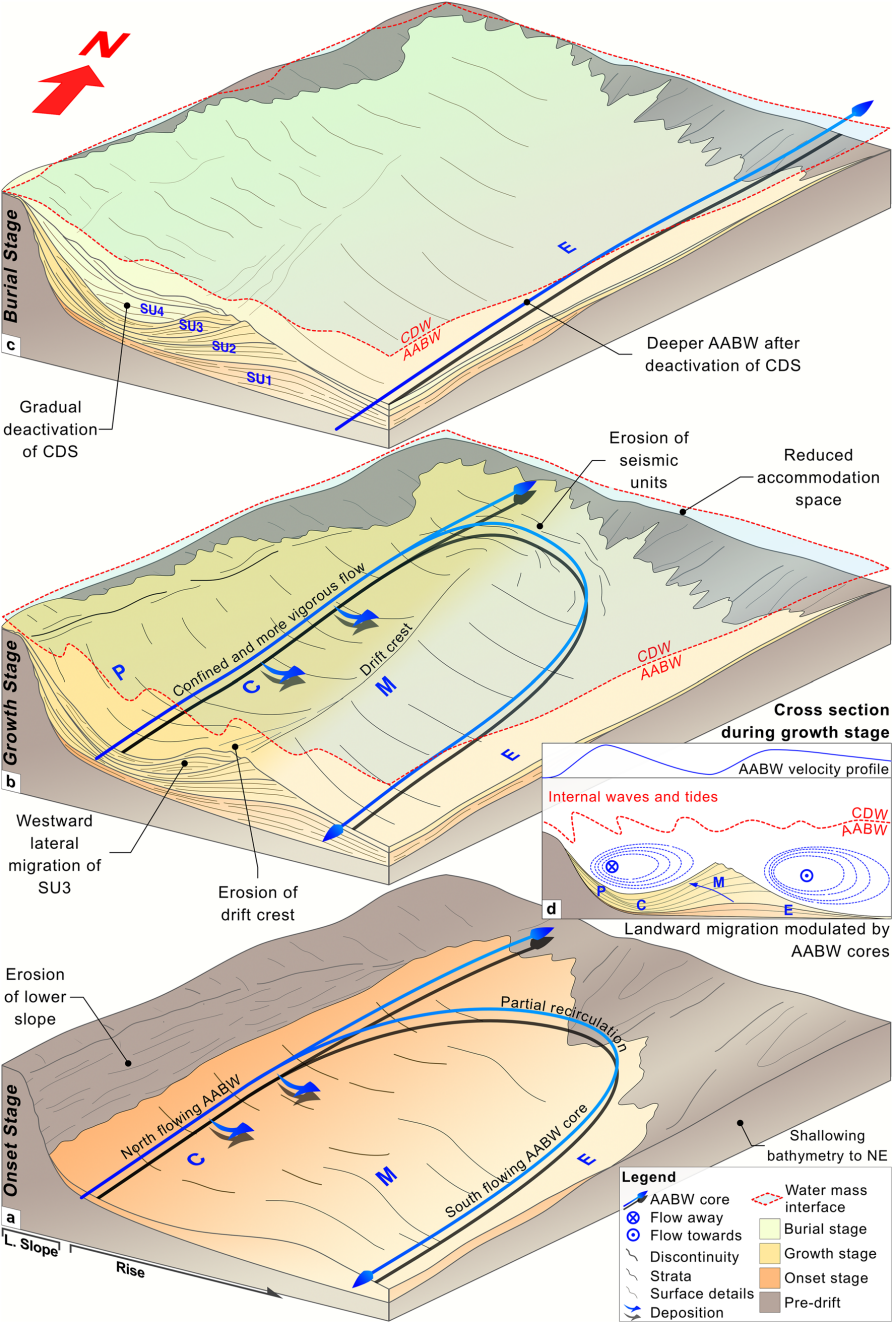
Conceptual models (**a**–**c**) depicting CDS evolutionary stages, AABW pathways, and AABW/CDW interface; cross-section diagram (**d**) of the upslope migration. The figure was generated using Pixelmator Pro 2.1.3 Coral (https://www.pixelmator.com/pro/).

The depositional and erosional features form a buried CDS whose onset and cessation are respectively marked by discontinuities D1 and D5. Each of the described seismic units thin to the northeast so the succession forms a wedge against a bathymetric high interpreted as a Cretaceous mixed turbidite-contourite system that formed in the distal part of the Colorado Basin (Figs. 1 and 2E)^5^. A distinctive stacking pattern appears both within and between the seismic units, revealing that the CDS developed over three evolutionary stages (Table 1 and Fig. 4A–C) which are described below.

I. The *Onset Stage* corresponds to SU1 (~ 34–25 Ma). SU1 is aggradational in all sectors. It consists of the plastered drift (P) against the lower slope, and a shallow mound, M, between C and E (Fig. 4A).

Within the Argentine basin, the AABW flowed northwards as a high-velocity western boundary current and significantly eroded the lower slope (Figs. 2A and 4A). Separate branches of this water mass, possibly deflected by bathymetric obstacles, generated drifts and channels^18^. At this time, the AABW/CDW interface occurred at about 2.5 km water depth^20^. From 33 to 29 Ma, the Drake Passage deepened to admit deep waters, as did the Tasman Strait from ~ 32–30 Ma^27^. This strengthened and deepened the Antarctic Circumpolar Current by 31–30 Ma, enhanced the AABW, and enabled the southward incursion of the Northern Component Water^20^. By ~ 27–24 Ma, the Atlantic meridional overturning circulation had become established^28^. A new deep water connection between the Argentine and Brazil Basins also increased circulation by the early Miocene^18^. Initiation of the plastered drift (P), contouritic channel (C), mounded drift (M), and adjacent erosional surface (E) during the onset stage, together with erosion of the lower slope indicate an efficient AABW with two local cores. One of these flowed along C, a relatively wide feature at this time, and the other generated E, a more localised feature (Figs. 2A, 3A, 4A).

II. The *Growth Stage* corresponds to SU2 and SU3 (~ 25–14 Ma). Aggradation is observed in P, upslope migration appears in C, and M shows prominent landward migration. In SU3, M and C shift in a landward direction.

From 21 to 15 Ma, the Drake Passage narrowed and thereby appears to have accelerated the AABW (Fig. 4B)^18,29^. From ~ 17 to 14 Ma, the Mid-Miocene Climatic Optimum caused the Atlantic meridional overturning circulation to rise to a shallower position in the water column. This in turn allowed Antarctic deep waters to migrate northward^30^. Exchange of bottom waters between the Argentine and Brazil basins likely occured by ~ 16 Ma^27^. At *ca.* 15 Ma, the margin underwent a period of vertical growth (the intermediate unit in Hernández-Molina et al., 2009)^20^ (Fig. 4B) likely associated with regional subsidence, global third-order highstand cycles^31^, and decreased bottom current activity due to the Mid-Miocene Climactic Optimum^15^. By the middle Miocene, the CDW had begun to separate into the NADW-derived LCDW and the UCDW. This caused the AABW/CDW interface to deepen to > 3.5 km. The NADW then flowed partially through the Central American Seaway into the Pacific, where it eventually joined the Antarctic Circumpolar Current (Fig. 4B,D)^20^. The significant growth of M in SU2 results from an invigorated AABW between ~ 21 and 15 Ma. The landward shift observed in C and M during SU3 may reflect weaker bottom currents during the Mid-Miocene Climactic Optimum as well as the deeper AABW/CDW interface. The drift crest of M and the uppermost boundary of P show truncation at ~ 3400 m water depth (Fig. 3A). This could represent more energetic secondary oceanographic processes (e.g., internal waves/tides) along the deeper AABW/CDW interface^11,32^. Deposition in P and M as well as minor aggradation in C reflect periods when the AABW was less energetic. Periodically invigorated bottom currents result in the development of the major bounding discontinuities between the seismic units^9,10^. Throughout the growth stage, E continues to show no signs of deposition.

III. The *Burial Stage* corresponds to SU4 (~ 14–7 Ma). Sub-unit a records relatively subtle lateral migration in M as C widens and shallows. Sub-unit b exhibits aggradation (Fig. 3A).

The burial stage coincides with shifts in ocean circulation possibly related to Miocene glaciation (Mi4), regression (Ser3), and a permanent eastern Antarctic ice-sheet^33^. Shallowing and closure of the Central American Seaway by ~ 6 Ma increasingly redirected the NADW into the South Atlantic thereby enhancing intermediate and deep water currents and causing AABW depocenters to deepen^20,27^. Gruetzner et al. (2012)^15^ explains that extensive erosion acompanied this changing oceanographic regime, resulting in the irregular discontinuity AR7 (Table 1). During the burial stage, C is gradually infilled as the CDS deactivates. The irregular D5 discontinuity, which truncates SU4 and laterally connects to E, represents the cessation of the CDS as the new oceanographic regime is established.

### Lateral migration of large sedimentary bodies in deep-marine systems

The asymmetric shape and internal sedimentary stacking pattern of the mounded drift (M) could share some similarities with asymmetric channel-levee systems^9,34^ or, channel-levee drifts from a mixed turbidite-contourite system^4,5,35^. However, in the absence of an observed feeder channel (or submarine canyon), and given the clear alongslope orientation and lateral continuity of the depositional and erosional features along the continental rise (rather than in the downslope orientation typically assumed by mixed turbidite-contourite systems), we consider here a pure Contourite Depositional System (CDS) and follow Gruetzner et al. (2012)^15^ in interpreting M as a large asymmetric mounded drift, because it meets criteria listed in Hernández-Molina et al. (2008)^36^. The drift resembles modern drifts offshore of South Africa^37^ or along the Mozambique Channel^11^. The M feature also resembles other, coeval, buried asymmetric mounded drifts found further south along the Patagonian margin^18^. Similarities include a closely related sedimentary stacking pattern and an erosive side, in this case, the surface E, away from which the drift’s crest migrates.

The generation of this CDS suggests a water mass (AABW) with two main cores. One core flows northward along C to erode the foot of the lower slope on its landward side and deposit sediment laterally on its basinward side (Fig. 4A)^9^. A second core flows in the opposite direction (southward) along the rise. The M feature forms between the two cores in a localised low velocity region similar to the Greater Antilles Outer Ridge^36^. The core along C forces M to migrate upslope and the southward flowing core erodes the distal flank of M to generate E (Fig. 4B)^18^. The northward-flowing AABW core is partially deflected by the bathymetric high to the northeast. This may occur due to the aforementioned Cretaceous mixed turbidite-contourite system depocenter (Figs. 2E, 4A–C)^5^ and/or because of bathymetric relief linked to the Ventana Transfer situated along the northern terminus of the drift (Fig. 1). The latter possibility follows an idea proposed by Hernández-Molina et al. (2010)^18^, whereby the northward flowing AABW was partially deflected by bathymetric relief associated with the Colorado Transfer (Fig. 1). This deflection resulted in a southward flowing AABW core which generated part of an asymmetric mounded drift coeval with the CDS described in this study, to the south of the El Austral Seamount (Figs. 1 and 3B). Partial rotation of the current first causes the truncation observed towards the northern edge of the drift and then generates E during the formation of the CDS (Figs. 2E, 3B and 4B). Deepening of the AABW/CDW interface around the middle Miocene confined the AABW below ~ 3.5 km water depth. This confinement may have enhanced the partial deflection of AABW due to reduced flow space between the interface and bathymetric high.

Overall, a complex bottom current regime controlled this CDS’s evolution. The large lateral migration observed in M is comparable in scale with large clinoform progradations. Furthermore, the internal sedimentary stacking of M appears similar to a transgression^24^. In SU2, the southward-flowing AABW core modulates the growth of M, causing it to migrate westward. This process resembles that of other asymmetric drifts described along the margin (Fig. 4D)^15,18^. The southward flow also maintains E as an area of non-deposition. The lateral upslope migration of M increasingly confines C, and by SU3, exhibits a westward shift into C. In contrast to other giant drifts in the region however, M shows no vertical growth^18^. The deeper AABW/CDW interface at this time appears to have imposed a vertical height limit on the CDS similar to that proposed for the Zambezi drift^11^. Secondary oceanographic processes, such as internal waves at this location, may cause enhanced erosion of the steep basinward flank of M^8^. Interestingly, these internal waves may represent a boundary similar to the ‘wave base’ in conventional progradational settings (Fig. 4D). The enhanced erosion may cause decreasing reflection gradients within M as accommodation space beneath the interface declines and the CDS deactivates (Fig. 4C).

## Conclusion

This study describes a contourite depositional system that exhibits the upslope lateral migration of large sedimentary bodies from Oligocene to middle Miocene times. The migration occurred due to global oceanographic changes and local bottom current processes and appears to have some similarities to major ‘clinoform’ progradations. The findings presented here carry implications for sedimentary basin analysis and paleoceanographic reconstructions.

## Data Availability

The data presented in this manuscript are subject to a non-disclosure agreement and therefore cannot be deposited in a repository.
